# Case series of total endovascular repair of the aortic arch with a modular inner-branched stent-graft system: first-in-man experience

**DOI:** 10.1093/bjs/znad070

**Published:** 2023-04-20

**Authors:** Hongpeng Zhang, Dan Rong, Feng Liu, Yangyang Ge, Ren Wei, Yating Zhu, Jiang Xiong, Wei Guo

**Affiliations:** Department of Vascular and Endovascular Surgery, Chinese PLA General Hospital, Beijing, China; Chinese PLA Medical School, Beijing, China; Department of Vascular and Endovascular Surgery, Chinese PLA General Hospital, Beijing, China; Department of Vascular and Endovascular Surgery, Chinese PLA General Hospital, Beijing, China; Department of Vascular and Endovascular Surgery, Chinese PLA General Hospital, Beijing, China; Department of Vascular and Endovascular Surgery, Chinese PLA General Hospital, Beijing, China; Department of Vascular and Endovascular Surgery, Chinese PLA General Hospital, Beijing, China; Department of Vascular and Endovascular Surgery, Chinese PLA General Hospital, Beijing, China; Chinese PLA Medical School, Beijing, China; Department of Vascular and Endovascular Surgery, Chinese PLA General Hospital, Beijing, China; Chinese PLA Medical School, Beijing, China

## Introduction

Open surgery of aortic arch pathologies is associated with mortality and postoperative complications, primarily because of the requirement for aortic cross-clamping and hypothermic circulatory arrest^1,2^. Since the first procedure was reported in 1999^3^, endovascular aortic arch repair techniques have been booming owing to the pursuit of minimally invasive treatment. The inner-branched stent-graft has now become the mainstream endovascular solution for reconstructing the branch vessels of the aortic arch^4–6^. A non-customized modular inner-branched stent-graft system was designed to reconstruct the aortic arch and preclinical study^7^ of this device has finished. The aim of this work was to carry out an IDEAL (Idea, Development, Exploration, Assessment and Long-term follow-up) surgical innovation stage 1 proof-of-concept study to evaluate the safety and feasibility of the stent-graft system (which has yet to receive regulatory approval for widespread clinical use) in a small group of highly selected patients.

## Methods

### Study design

This prospective first-in-man study enrolled the first 15 patients receiving endovascular aortic arch repair using the novel stent-graft at Chinese PLA General Hospital from February 2019 to January 2022. The protocol of this study was registered with ClinicalTrials.gov (NCT04764370) and approved by the Institutional Review Board of Chinese PLA General Hospital. This study was performed under the supervision of National Medical Products Administration of China. Regulatory approval for the device is being sought.

### Patients

The multidisciplinary team assessed 49 consecutive patients with aortic arch aneurysms, of whom 15 were included in this study. Of the 34 patients excluded, 21 underwent open surgery because of acceptable surgical risk, 7 had a maximum ascending aortic diameter exceeding 44 mm, 3 had a severely calcified aorta, and 3 had a high atherosclerotic burden. Written informed consent was obtained from each patient after discussion of the risks, benefits, and alternatives. All patients were specifically informed that this was the first clinical application of the novel device.

### Intervention

This novel stent-graft system (Endonom Medtech, Hangzhou, China) consists of one proximal main body, one distal main body, and two bridging covered stents (*Fig. S1*).

All procedures were performed under general anaesthesia by a single operator with over 20 years’ experience in endovascular aortic procedures. The proximal main body was delivered to the ascending aorta over the stiff wire, and deployed proximal to the innominate artery under right ventricular pacing (180–220 beats/min) or intravenous deliberate hypotension (systolic BP 90 mmHg or less) (*Fig. S2a*). Bridging covered stents were then deployed to reconstruct the innominate artery and the left common carotid artery successively (*Fig. S2b*). Finally, the distal main body was released to isolate the aortic arch pathologies (*Fig. S2c*).

## Results

A total of 15 patients (1 woman) were enrolled. The median age was 68 (i.q.r. 64–73) years. Generalized atherosclerotic vascular disease was common in participants, 7 patients had coronary artery disease, 7 patients cerebrovascular disease, and 5 patients had undergone a coronary bypass or percutaneous intervention previously (*Table S1*).

Among all 15 patients, a fusiform aneurysm (46.7 per cent) was the main type of pathology; other types included penetrating aortic ulcers (13.3 per cent), pseudoaneurysm (6.7 per cent), saccular aneurysms (20 per cent), and postdissection aneurysms (13.3 per cent) (*Table S2*).

Thirteen procedures (86.7 per cent) were performed electively, whereas two (13.3 per cent) were urgent operations. The median procedure time was 246 (i.q.r. 210–360) min, and the median duration of fluoroscopy was 50 (42.5–65.5) min. Median duration of postoperative hospital stay was 6 (5–9.5) days, including a median ICU stay of 2 (0–4) days. (*Table S3*).

**Fig. 1:**
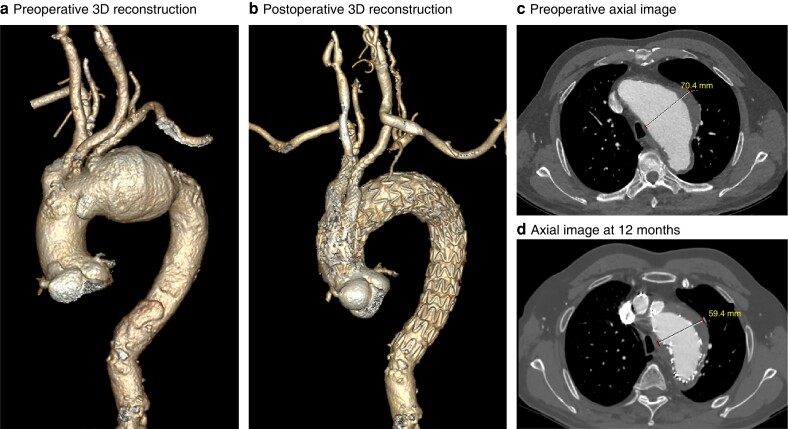
Preoperative and postoperative three-dimensional reconstructions and axial images **a** Preoperative and **b** postoperative three-dimensional (3D) reconstructions. **c** Preoperative and **d** follow-up axial images showing shrinkage of the aneurysm by 12 months after operation.

Technical success was achieved in all 15 patients. Median follow-up time was 368 (i.q.r. 189.5–916.5) days. The combined perioperative stroke rate was 13.3 per cent (2 of 15). One patient died from cerebral haemorrhage 5 days after stent-graft implantation because of a coagulation disorder. This patient was also diagnosed with acute renal insufficiency after operation. One patient had a minor stroke immediately after operation and recovered after 7 days. One patient suffered iliac artery rupture during the procedure. Paraplegia occurred in one patient immediately after the operation, with recovery after 5 days. Fourteen patients underwent at least one postoperative computed tomography angiography (CTA) (*Tables S4* and *S5*, and *Fig. 1*).

## Discussion

In this first-in-human study, the stent-graft system was found to be feasible and safe for treatment of patients with aortic arch disease. Although operations were performed in patients at high surgical risk, the perioperative mortality rate (1 of 15) was acceptable. Based on strict preoperative aortic anatomical screening, technical success was achieved in all patients.

Cerebrovascular events are one of the major concerns of aortic arch repair in both open surgery and endovascular repair. Two patients had a stroke in this case series; one had a non-disabling ischaemic stroke, and the other had a delayed fatal cerebral haemorrhage. The symptoms of non-disabling stroke presented immediately after operation, and the stroke was suspected to be caused by atheromatous debris or air embolism. The fatal cerebral haemorrhage occurred 5 days after operation because of consumptive coagulopathy possibly resulting from a large aneurysm diameter (96.3 mm). The stroke rate was acceptable (13.3 per cent) compared with the results for currently available aortic arch branched devices^7,8^. Technical points to lower the stroke rate included careful patient selection, minimizing the duration of operation, and wire manipulation.

The main reasons for reintervention after endovascular aortic arch repair were endoleaks and access complications. In the present study, cervical bleeding occurred in four patients. Additionally, there was one case of intraoperative iliac artery bleeding owing to the oversize of the delivery sheath; this was immediately repaired with an 8 × 150-mm covered stent (W. L. Gore & Associates, Newark, DE, USA). No endoleak required reintervention in this study. The relatively short follow-up and small sample size may be one of the reasons. Another reason may be an adequate healthy landing zone in the ascending aorta.

In this study, nine patients with aneurysms starting from zone 2 underwent total endovascular aortic arch repair because of an inadequate healthy landing zone in zone 1. In these patients, the entire healthy zone 0 served as landing zone, which would be beneficial to a low rate of type I endoleak.

The main limitation of this study is that all procedures were performed in selected patients by a single operator. Therefore, generalization of the outcomes should be undertaken with caution. The further multicentre study should be performed only in high-volume centres with extensive experience in the management of complex aortic disease by both open and endovascular treatment. Additionally, because this was a prospective first-in-man study, there was no control group of patients who underwent open surgery or endovascular repair using other available devices.

The results of the present study have demonstrated the technical feasibility and safety of endovascular aortic arch repair in patients at high surgical risk using this non-customized modular inner-branched stent-graft system. The stroke and reintervention rates were favourable. An IDEAL stage IIa study is planned to further evaluate the efficacy and obtain additional data on this novel aortic arch stent-graft.

## Supplementary Material

znad070_Supplementary_Data

## Data Availability

Because of the sensitive nature of the data collected for this study, requests to access the data set from qualified researchers trained in human subject confidentiality protocols may be sent to guoweiplagh@sina.com.
