# Supplementary material for: Case series of total endovascular repair of the aortic arch with a modular inner-branched stent-graft system: first-in-man experience
Source: Br J Surg. 2023 Apr 20;110(9):1084–6. doi: 10.1093/bjs/znad070 (PMC10416682; doi:10.1093/bjs/znad070)
Supplement: znad070_Supplementary_Data [file znad070_supplementary_data.docx]

**Case-series of total endovascular repair of the aortic arch with a modular inner branched stent-graft system: first-in-man experience**

Hongpeng Zhang^1,2,†^, Dan Rong^1,^^†^, Feng Liu^1^, Yangyang Ge^1^, Ren Wei^1^, Yating Zhu^1^, Jiang Xiong^1,2^ and Wei Guo^1,2,*^

^1^Department of Vascular and Endovascular Surgery, Chinese PLA General Hospital, Beijing, China

^2^Chinese PLA Medical School, Beijing, China

† The first two authors contributed equally to this work.

**Corresponding author：**Wei Guo, Department of Vascular and Endovascular Surgery, Chinese PLA General Hospital, 28 Fuxing Road, Beijing 100853, China (E-mail: [guoweiplagh@sina.com)](mailto:guoweiplagh@sina.com)) **ORCID ID**：0000-0001-6212-8390

**Supplementary Materials - Index**

| **Supplementary Methods** |  |
| --- | --- |
| Indications for intervention | *pag. 3* |
| Details of device | *pag. 3* |
| Details of procedures | *pag. 4* |
| Follow-up methods | *pag. 5* |
| Statistical Analysis | *pag. 6* |
| **Supplementary Results** |  |
| Additional procedural details | *pag. 7* |
| Additional details of outcomes | *pag. 7* |
| **Supplementary Figures and Tables** |  |
| Figure S1 | *pag. 9* |
| Figure S2 | *pag. 9* |
| Table S1 | *pag. 10* |
| Table S2 | *pag. 12* |
| Table S3 | *pag. 11* |
| Table S4 | *pag. 13* |
| Table S5 | *pag. 14* |
| **References** | *pag. 16* |

**Supplementary Methods**

**Indications for intervention and anatomical requirement**

Indications for intervention were a maximum aortic diameter > 55 mm, pending ruptured aneurysms (regardless of aneurysm size), or rapid increase of aortic diameter (> 10 mm in 1 year). The anatomical requirements for the proximal landing zone in the ascending aorta were a diameter between 24 mm and 44 mm and a length of at least 50 mm between the sinotubular junction and the proximal edge of the innominate artery. Additional anatomical requirements include innominate artery diameter between 7 mm and 24 mm and at least 20 mm in length; left common carotid artery (LCCA) diameter between 7 mm and 24 mm; iliofemoral access diameter of at least 7 mm. All patients with grade IV or V aortic atheroma were excluded^1^.

**Details of device**

The proximal main body has a length of 50 or 60 mm, and was loaded in a pre-curved 22- or 24-Fr (main body diameter ≥ 40 mm) hydrophilic delivery system to ensure self-alignment of the device in the ascending aorta. There were three tunnels inside the proximal main body: two internal branches for reconstruction of supra-aortic arteries and one main tunnel for connection of distal main body to the descending aorta. The gutters between the main tunnel and two inner branches were prophylactically sealed by a sutured membrane to prevent endoleak (Fig. S1c). The internal branches were 20 mm in length and 10 (main body diameter ≤36 mm) or 12 (main body diameter >36 mm) mm in diameter. In surgical planning measurement, the whole length of proximal main body was regarded as landing zone. Both the maximum and minimum oversize of the landing zone were required to be less than 20%. The proximal diameter of bridging covered stents were 13 mm for connecting 12-mm inner branches and 11 mm for 10-mm inner branches. The distal diameter ranged from 8 to 26 mm and the length from 60 to 140 mm. The bridging covered stent was loaded in a 12- or 14-Fr (stent diameter ≥18 mm) delivery system. The distal main body was loaded in a pre-curved 22-Fr hydrophilic delivery system.

**Details of procedures**

Before endovascular procedures, a left subclavian artery (LSA) to LCCA bypass was established through a left cervical incision. Endovascular procedures were performed after reaching an activated clotting time (ACT) of over 250 seconds. The ACT was measured every hour and maintained between 250s to 350s by intermittent heparin infusion. A total of four arterial accesses were needed in these procedures: femoral access to deploy two main bodies, right brachial or right carotid access to reconstruct the innominate artery, left carotid access to reconstruct LCCA, and left brachial access to embolize LSA.

Firstly, a pigtail catheter (Cordis, Miami Lakes, FL, USA) was advanced into the left ventricle for angiogram through femoral access. A Lunderquist stiff wire (COOK Medical, Bloomington, IN, USA) was advanced into the left ventricle under the protection of a pigtail catheter. The proximal main body was then delivered to ascending aorta over the stiff wire and deployed proximal to the innominate artery (Fig. S2a) under right ventricular pacing (180-220 beats/min) or intravenous deliberate hypotension (systolic pressure ≤90 mmHg).

After the deployment of proximal main body, a 12- or 14-Fr 55-cm Dryseal sheath (W.L. Gore, Flagstaff, AZ, USA) and a 12-Fr 40-cm Flexor sheath (COOK Medical, Bloomington, IN, USA) were introduced through right brachial access and left carotid access respectively. Two Supra Core guide wires (Abbott, Chicago, IL, USA) were then placed in the internal branches of proximal main body. Before the placement of bridging covered stents, two parallel 12×40-mm balloons were inflated simultaneously to re-confirm the correct catheterization. When deflating the two balloons, sheaths were advanced into the internal branches using balloon assisted tracking technique. Bridging covered stents were deployed through these two sheaths to reconstruct the innominate artery and the LCCA successively (Fig. S2b).

To avoid entanglement with the 2 bridging covered stents, the Lunderquist stiff wire (COOK Medical, Bloomington, IN, USA) was withdrawn to the descending aorta and then re-advanced to the aortic root inside a rotating pigtail catheter (Cordis, Miami Lakes, FL, USA). The distal main body was delivered to the desired position through the stiff wire and then released to isolate the aortic arch pathologies. After ballooning the internal branches, completion angiography was performed to detect any endoleak (Fig. S2c). At the end of procedures, protamine was used to neutralize residual unfractionated heparin with a protamine:heparin ratio of 0.5.

**Follow-up methods**

This case series has been reported in line with the IDEAL Framework & Recommendations and reporting standards for thoracic endovascular aortic repair^2^. Clinical examinations and CTA test with 0.625 mm thickness were performed before discharge, then at 1, 6, and 12 months and annually thereafter. Three months of dual antiplatelets after operation and lifelong use of aspirin are advised. Technical success was defined by successful introduction and deployment of the components in the absence of surgical conversion or mortality, type I or III endoleak, or graft obstruction within 24 hours of the procedure^2^. Clinical success was confirmed without any of the following: death as a result of treatment or as a result of the original pathology that was treated; type I or III endoleak, infection or aortic thrombosis; aneurysm expansion or rupture; conversion to open repair; or failure to arrest the original pathologic process or causing a new thoracic aortic pathology as a result of the intervention. Branch stenosis was defined as >50% stenosis on CTA or ultrasound. Aneurysm sac shrinkage was defined as at least a 5 mm decrease in maximum aortic diameter. All data were measured by two independent vascular surgeons with over five years of experience. Any serious adverse event was required to be reported to the ethics boards and regulatory agencies within 72 hours.

**Statistical Analysis**

Categorical variables are presented as percentages. Continuous variables are presented as medians with inter-quartile ranges (Q1–Q3). A value of p < 0.05 was used to determine statistical significance.

**Supplementary Results**

**Additional procedural details**

All operations were performed under general anesthesia. The median volume of contrast media injected was 180 (IQR, 160–200) mL. LCCA–LSA prosthetic bypass was performed simultaneously in 8 patients (53.3%) (Table 2). The reasons for 7 patients not undergoing bypass included 2 left vertebral arteries (LVAs) arising from the aortic arch, 2 occluded LVAs, 1 severe stenosis of LVA, 1 occluded left internal carotid artery (reconstruction of IA and LSA), and 1 hypogenetic LVA. The proximal main body was deployed under cardiac output reduction using rapid ventricular pacing in early experience group and intravenous vasodilator in late experience group. Moreover, retrograde access for the IA stenting was the right common carotid artery in early experience group and the right brachial artery in late experience group.

**Additional details of outcomes**

No secondary intervention was needed. No aortic arch aneurysm-related death or aneurysmal diameter evolution was observed during follow-up. Six patients (40%) presented with shrinkage of aneurysmal sac, while eight patients (53.5%) presented with a stable aneurysm sac. During follow-up, all endografts and branches remained patent with no stent migration. A 60% asymptomatic anastomotic stenosis of LCCA–LSA bypass was observed at 6 months. One type II endoleak self-sealed within 6 months. No new-onset endoleak was observed. One iliac rupture was repaired with covered stent (Viabahn 8×150 mm; W.L. Gore & Associates, Newark, DE, USA) during the procedure.

The early experience group and late experience group were comparable in terms of comorbidities. The median procedure time (315 [237–406.5] minutes vs 240 [198–261] minutes, p = 0.045) and fluoroscopy time duration (65.5 [58.3–68.5] minutes vs 42 [41–44] minutes, p < 0.001) decreased in the late group. There was no statistically significant difference in median volume of contrast media, endoleak, early mortality, sac shrinkage rate, and postoperative hospital.

**Supplementary Figures and Tables**

**
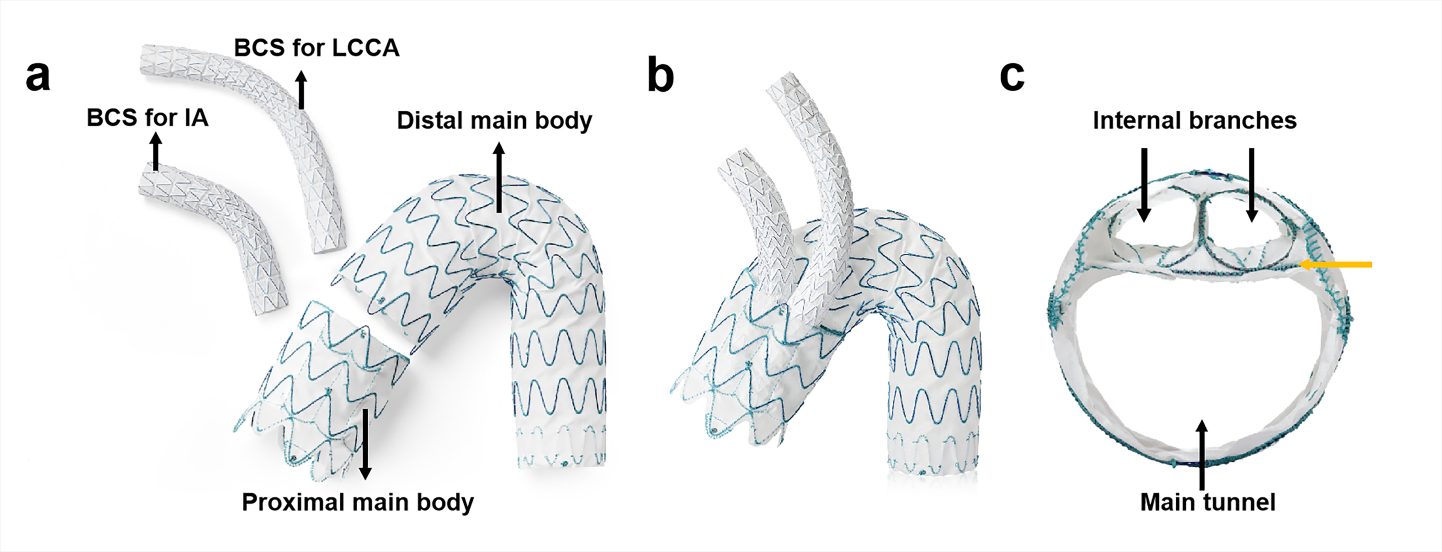
**

**Fig. S1 a** Modules of the non-customized stent-graft. **b** Configuration of assembled modular aortic arch stent-graft. **c** Internal view of proximal main body, yellow arrow indicates the sutured membrane to prevent endoleak. BSC, bridging covered stent; IA, innominate artery; LCCA, left common carotid artery.

**
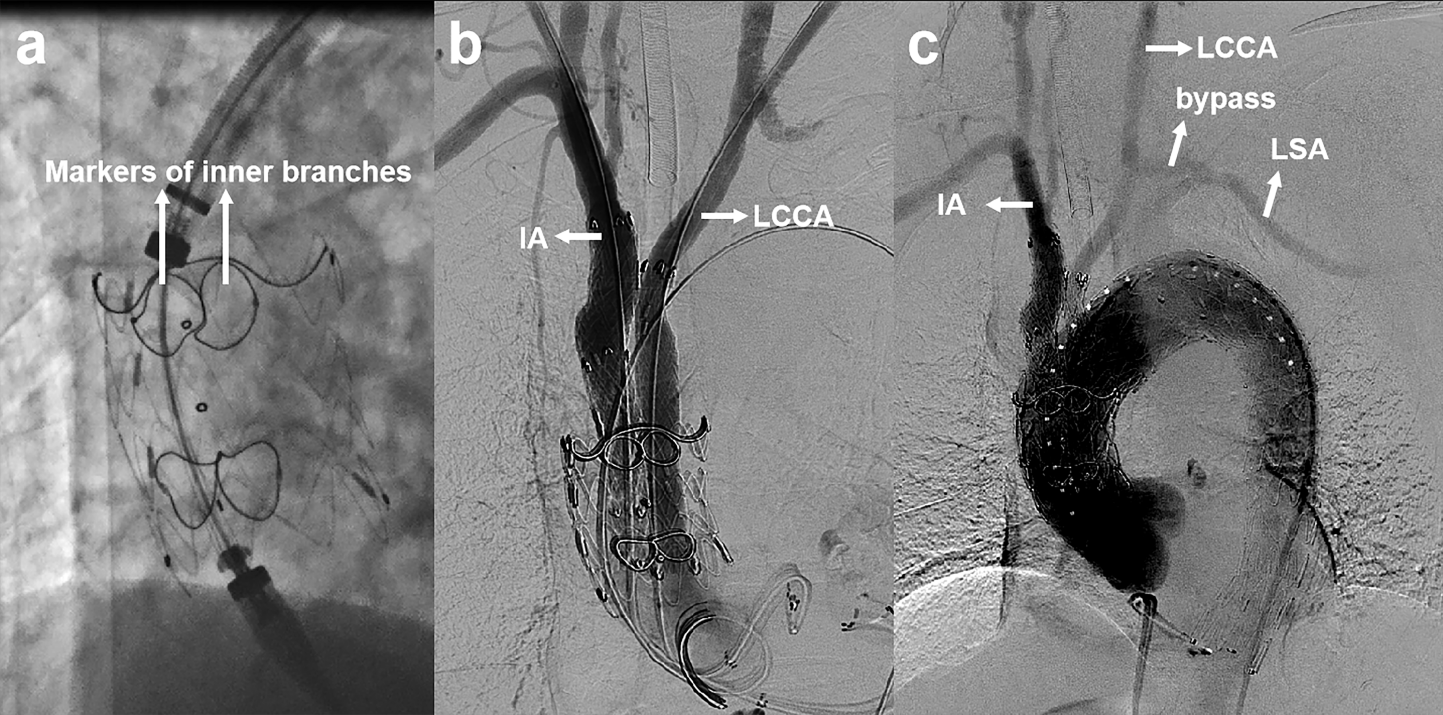
**

**Fig. S2 a** The implantation of proximal main body. **b** Reconstruction of IA and LCCA. **c** Reconstruction of total aortic arch. IA, innominate artery; LCCA, left common carotid artery; LSA, left subclavian artery.

**Table S1.** **Baseline characteristics**

| **Patients** | **n=15** |
| --- | --- |
| **Age (years)** | 68 (64-73) |
| **Male** | 14 (93.3) |
| **EuroSCORE II** | 3.12 (2.42-3.93) |
| **NYHA classification** |  |
| I | 8 (53.3) |
| II | 4 (26.7) |
| III | 3 (20.0) |
| IV | 0 (0.0) |
| **ASA class 3 or 4** | 6 (40.0) |
| **Smoking** | 13 (86.7) |
| **Hypertension** | 13 (86.7) |
| **Diabetes mellitus** | 2 (13.3) |
| **Dyslipidemia** | 8 (53.3) |
| **COPD** | 3 (20.0) |
| **Chronic renal failure** | 3 (20.0) |
| **Coronary artery disease** | 7 (46.7) |
| **Previous coronary artery bypass graft** | 2 (13.3) |
| **Previous percutaneous coronary intervention** | 3 (20.0) |
| **Cerebrovascular disease** | 7 (46.7) |
| **Previous TEVAR** | 2 (13.3) |

NYHA, New York Heart Association; ASA, American Society of Anesthesiologists; COPD, chronic obstructive pulmonary disease; TEVAR, thoracic endovascular aortic repair.

**Table S3. Procedural details**

|  | **All cohort (n=15)** | **Early experience group (n=8)** | **Late experience group (n=7)** | ***P* value** |
| --- | --- | --- | --- | --- |
| **Emegency operation** | 2 (13.3) | 0 (0) | 2 (28.6) | 0.2 |
| **Procedure time, minutes** | 246 (210-360) | 315 (237-406.5) | 240 (198-261) | 0.045 |
| **Fluoroscopy time, minutes** | 50 (42.5-65.5) | 65.5 (58.3-68.5) | 42 (41-44) | <0.001 |
| **Volume of contrast media, mL** | 180 (160-200) | 190 (160-207.5) | 180 (165-200) | 0.939 |
| **Postoperative hospital stay, days** | 6 (5-9.5) | 6.1 (5.8-10.8) | 5.3 (5-8.5) | 0.114 |
| **Intensive care unit stay, days** | 2 (0-4) | 2.5 (0-3.5) | 2.00 (0-4) | 0.613 |
| **LCCA-LSA bypass** | 8 (53.3) | 4 (50.0) | 4 (57.1) | 0.782 |
| **Access for innominate artery reconstruction** |  |  |  | <0.001 |
| Right brachial artery | 7 (46.7) | 0 (0) | 7 (100) |  |
| Right common carotid artery | 8 (53.3) | 8 (100) | 0 (0) |  |
| **Blood pressure lowering** |  |  |  | <0.001 |
| Rapid-pacing | 8 (53.3) | 8 (100) | 0 (0) |  |
| Intravenous hypotension | 7 (46.7) | 0 (0) | 7 (100) |  |

LCCA, left common carotid artery; LSA, left subclavian artery.

**Table S2. Aortic** **anatomical characteristics and device selection**

| **Patient** | **Type of aortic pathology** | **Beginning zone of lesion** | **End zone of lesion** | **Maximum diameter of aneurysms (mm)** | **Length of ascending aorta (mm)** | **Diameter of ascending aorta (mm)** | **Length of innominate artery (mm)** | **Diameter of innominate artery (mm)** | **Diameter of LCCA (mm)** | **Proximal main body (mm)** | **Proximal oversize (%)** | **Bridging covered stent for innominate artery (mm)** | **Bridging covered stent for LCCA (mm)** | **Distal main body (mm)** |
| --- | --- | --- | --- | --- | --- | --- | --- | --- | --- | --- | --- | --- | --- | --- |
| **1** | Penetrating aortic ulcer | Z1 | Z3 | 41.5 | 62.30 | 40.8 | 34.6 | 16.9 | 9.1 | 46-12-50 | 11.3 - 18.2 | 13-13-70 13-20-80 | 13-13-80 13-10-100 | 44-38-200 |
| **2** | Pseudoaneurysm | Z1 | Z3 | 53.1 | 71.30 | 29.8 | 39 | 12.3 | 6 | 34-10-50 | 12.4 - 17.3 | 11-13-100 | 11-10-100 | 32-26-200 |
| **3** | Penetrating aortic ulcer | Z2 | Z4 | 54.8 | 58.70 | 34.7 | 32 | 13.2 | 7.6 | 38-12-50 | 8.7 - 15.3 | 13-16-120 | 13-9-120 | 34-28-200 |
| **4** | Saccular aneurysm | Z1 | Z3 | 40.3 | 57.20 | 38.8 | 48.3 | 15.2 | 9.6 | 42-12-50 | 7.6 - 18.5 | 13-13-80 13-18-100 | 13-10-120 | 38-32-200 |
| **5** | Post-dissection aneurysm | Z0 | Z3 | 59.1 | 59.60 | 42.5 | 30.5 | 13.1 | 6.8 | 44-12-50 | 3.4 - 17.8 | 13-16-100 | 13-13-160 | 40-32-200 |
| **6** | Fusiform aneurysm | Z0 | Z4 | 66.0 | 62.10 | 41.1 | 47 | 16.8 | 9.2 | 44-12-50 | 6.6 - 19.1 | 13-18-100 | 13-13-70  13-12-100 | 40-30-200 |
| **7** | Saccular aneurysm | Z2 | Z4 | 50.9 | 55.40 | 39.2 | 53 | 17 | 9.6 | 42-12-50 | 6.7 - 16.9 | 13-18-100 | 13-12-140 | 38-30-200 |
| **8** | Ruptured fusiform aneurysm | Z2 | Z4 | 138.0 | 63.50 | 43.4 | 51 | 20 | 8.5 | 46-12-60 | 5.7 - 19.7 | 13-22-120 | 13-12-120 | 40-30-200 |
| **9** | Fusiform aneurysm | Z2 | Z3 | 56.9 | 62.30 | 32.5 | 30 | 10.9 | 7.2 | 34-10-60 | 4.4 - 18.8 | 11-12-120 | 11-8-120 | 30-24-200 |
| **10** | Fusiform aneurysm | Z2 | Z3 | 58.1 | 69.20 | 44 | 40.5 | 19.4 | 11.7 | 48-12-60 | 8.3 - 15.5 | 13-20-120 | 13-12-140 | 44-34-200 |
| **11** | Impending ruptured fusiform aneurysm | Z0 | Z3 | 96.3 | 66.20 | 35.4 | 38.5 | 13.6 | 7.5 | 38-12-60 | 6.8 - 16.3 | 13-16-120 | 13-8-120 | 34-30-200 |
| **12** | Fusiform aneurysm | Z2 | Z3 | 52.2 | 60.20 | 35.8 | 45.8 | 12.8 | 8.9 | 38-12-50 | 5.8 - 18.4 | 13-16-100 | 13-10-120 | 34-30-200 |
| **13** | Saccular aneurysm | Z2 | Z3 | 49.8 | 57.90 | 36.5 | 57.9 | 13.6 | 8.4 | 40-12-50 | 8.6 - 13.8 | 13-18-100 | 13-10-100 | 36-28-200 |
| **14** | Post-dissection aneurysm | Z2 | Z4 | 60.3 | 63.40 | 39.5 | 53.5 | 16.2 | 8.1 | 44-12-60 | 10.2 - 14.5 | 13-18-120 | 13-10-120 | 42-38-200 |
| **15** | Fusiform aneurysm | Z2 | Z4 | 59.3 | 65.80 | 40.2 | 29.7 | 14.4 | 6.8 | 44-12-60 | 8.6 - 17.1 | 13-16-100 | 13-8-120 | 42-38-200 |

**Table S4. Outcomes of patients**

| **Patient** | **Age (years)** | **Sex** | **Follow-up (days)** | **Mortality** | **Stroke** | **Endoleak** | **Paraplegia** | **Acute renal injury** | **Estimated blood loss >1 L** | **Myocardial infarction** | **Respiratory failure** | **New-onset dialysis** | **Bowel ischemia** | **Access complication** | **Δ Maximum diameter of aneurysm (mm)** |
| --- | --- | --- | --- | --- | --- | --- | --- | --- | --- | --- | --- | --- | --- | --- | --- |
| **1** | 74 | male | 1103 | No | No | No | No | No | No | No | No | No | No | No | -5.3 |
| **2** | 59 | male | 1091 | No | No | No | No | No | No | No | No | No | No | Cervical hematoma | -6.2 |
| **3** | 58 | male | 1115 | No | No | No | No | No | No | No | No | No | No | No | -5.2 |
| **4** | 66 | male | 1096 | No | No | No | No | No | No | No | No | No | No | No | -3 |
| **5** | 53 | male | 742 | No | No | No | No | No | No | No | No | No | No | Cervical hematoma | 0.2 |
| **6** | 68 | male | 362 | No | No | No | Recovered 7 days after operation | No | No | No | No | No | No | No | -7.4 |
| **7** | 69 | male | 368 | No | No | Type II | No | No | No | No | No | No | No | No | -3.9 |
| **8** | 68 | male | 370 | No | No | No | No | No | No | No | No | No | No | Cervical hematoma | -32.6 |
| **9** | 73 | male | 365 | No | No | No | No | No | No | No | No | No | No | Iliac artery injury | -3.7 |
| **10** | 70 | male | 369 | No | No | No | No | No | No | No | No | No | No | No | -0.9 |
| **11** | 76 | male | 5 | Died of cerebral hemorrhage | Cerebral hemorrhage | No | No | Creatinine increased from 163.9 umol/L to 292.4 umol/L | No | No | No | No | No | Cervical hematoma | NA |
| **12** | 83 | female | 181 | No | No | No | No | No | No | No | No | No | No | No | -1.3 |
| **13** | 72 | male | 185 | No | Non-disabling stroke | No | No | No | No | No | No | No | No | No | 0.9 |
| **14** | 66 | male | 194 | No | No | No | No | No | No | No | No | No | No | No | -5.7 |
| **15** | 64 | male | 180 | No | No | No | No | No | No | No | No | No | No | No | 0 |

**Table S5. IDEAL checklist for IDEAL stage 1.**

| **STAGE 1** | **Item** | **Checklist Item for key IDEAL items** |  |
| --- | --- | --- | --- |
| Title and Abstract | 1a | Identify the technique or device in the title, including IDEAL Stage 1 or ‘first in human’ in the title or abstract. | Yes |
|  | 1b | Provide a structured summary of background, methods, results, and conclusions. | Yes. In the submission system. |
| **Introduction** |  |  |  |
| Background and objectives | 2a | Review of existing scientific literature, providing a clear explanation of the rationale for the new technique, including unmet clinical need. | Yes. Page 2, line 7-30. |
|  | 2b | Details of pre-clinical development of the technique, including assessment of risks of failure and analysis of efforts to avoid harm. | Yes. Page 2, line 25. |
| **Methods** |  |  |  |
| Design | 3 | Description of study design (e.g. case report or very small case series). | Yes. Page 2, line 38-43. |
| Participants | 4a | Transparent account of patient selection, with explicit detail about inclusion and exclusion criteria. | Yes. Page 3. line 4-12. Page 13, line 9-27. |
|  | 4b | Informed consent process described, including explanation of risks and acknowledgement of level of experience with technique/device. If informed consent is not obtained due to unplanned technique or modification, describe the discussion with the patient after the innovation. occurred. | Yes. Page 3. line 12-17. |
|  | 4c | Setting, location, and timeframe of when and where the novel technique was performed, including hospital characteristics and appropriate details regarding the operator/team (e.g. experience). | Yes. Page 2, line 37-43. Page 3, line 30-33. |
| Intervention | 5a | Clear and detailed description of the new technique/device, including necessary pre- and post-procedure care. | Yes. Page 3, line 22-51. Page 13-15. |
|  | 5b | Patient safety monitoring methods and safeguards. | Yes. Page 15, line 18-23. |
| Outcomes | 6 | Description of outcome measure(s) selected and how they were assessed, including patient reported outcome measures, if appropriate, utilising those measures that are standardised and validated, when available and applicable. When these are not available, provide rationale for the outcome measure(s) used. | Yes. Page 15, line 15-38. |
| **Results** |  |  |  |
| Baseline Data | 7 | Baseline demographic and clinical characteristics for each patient. Include how many patients were assessed for treatment and a description of which patients were included, excluded, or refused, and why (to be displayed in a flow diagram format, when appropriate). | Yes. Page 4, line 14-25. Page 11. Page 2 line 58 - Page 3 line 12. |
| Intervention | 8 | Technical feasibility of technique, including visual aids (e.g. photographs, videos, etc) when available. | Yes. Page 4, line 40- 54. Page 10. Page 16, line 8-29. |
| Outcomes | 9 | Appropriate clinical outcomes, including patient-reported outcome measures, when applicable. | Yes. Page 4, line 56-59. Page 16, line 34-58. |
| Harms | 10 | Transparent account of all harms or unintended effects reported for each patient. | Yes. Page 5, line 4-22. |
| **Discussion** |  |  |  |
| Stage End-Points | 11 | Author’s overall appraisal of the new technique, including discussion of risks and harms reported and suggestions to avoid them in future cases based on initial experience. | Yes. Page 5,6 |
| Conclusions | 12 | Conclusions and relevance, including plans to progress to future IDEAL stages, or plans to discontinue further research. | Yes. Page 7, line 4-15. |
| **Other Information** |  |  |  |
| Protocol | 13 | Please quote reference or DOI if a protocol was written in advance and made available. If a protocol was not made available, consider including as a supplement if the journal allows | Yes. Page 2, line 43-46. |
| Ethics | 14 | Reference to ethical approvals obtained, and independent oversight, if applicable | Yes. Page 2, line 45-51. |
| Funding | 15 | Sources of funding and support, role of funders, and other conflicts of interest | Yes. Page 7 |
| Regulatory Approvals | 16 | Regulatory approvals being sought or obtained (e.g. CE Marking, FDA approval, etc) including the date of approval, if applicable | Yes. Page 2, line 51-53. |

**References**

1. Gutsche JT, Cheung AT, McGarvey ML, Moser WG, Szeto W, Carpenter JP, *et al.* Risk factors for perioperative stroke after thoracic endovascular aortic repair. *Ann Thorac Surg*. 2007;**84**:1195-200; discussion 1200.
2. Chaikof EL, Blankensteijn JD, Harris PL, White GH, Zarins CK, Bernhard VM *et al.*; Ad Hoc Committee for Standardized Reporting Practices in Vascular Surgery of The Society for Vascular Surgery/American Association for Vascular Surgery. Reporting standards for endovascular aortic aneurysm repair. *J Vasc Surg.* 2002;**35**:1048-60.
